# Effect of Surface Treatments on Shear Bond Strength of Polyetheretherketone to Autopolymerizing Resin

**DOI:** 10.3390/dj7030082

**Published:** 2019-08-01

**Authors:** Kosuke Kurahashi, Takashi Matsuda, Yuichi Ishida, Tetsuo Ichikawa

**Affiliations:** Department of Prosthodontics & Oral rehabilitation, Tokushima University, Graduate School of Biomedical Sciences, 3-18-15 Kuramoto, Tokushima 770-8504, Japan

**Keywords:** polyetheretherketone (PEEK), shear bond strength, acrylic resin, clasp retainer

## Abstract

These days, new prosthodontic materials are appearing with the development of digitalization. Among these, the use of polyetheretherketone (PEEK) as the clasp of removable partial dentures has been proposed. The adhesive strength between the PEEK and acrylic resin influences the probability of denture fracture. To investigate the effect of PEEK surface treatments on the shear bond strength to acrylic resin, five surface treatment conditions of PEEK were analyzed: 1. no treatment; 2. ceramic primer application; 3. Al_2_O_3_ sandblasting; 4. Rocatec; and 5. Rocatec with ceramic primer application, comparing with a metal primer-treated Co-Cr alloy. Two kinds of autopolymerizing resin (Unifast II and Palapress Vario) were used as bonding materials. The specimens were evaluated to determine the bond strength. Rocatec treatment with ceramic primer application yielded the highest bond strengths (12.71 MPa and 15.32 MPa, respectively, for Unifast II and Palapress Vario). When compared to a metal primer-treated Co-Cr alloy, the bond strength of PEEK to Unifast II was similar, whereas it was about 60% of that to Palapress Vario. Rocatec treatment, combined with ceramic primer, showed the highest bond strength of PEEK to acrylic resin. Treatment of PEEK will enable its use as the clasp of removable dentures and the fixation of PEEK prostheses.

## 1. Introduction

Polyetheretherketone (PEEK) is an engineering plastic material that is widely applied in industrial products due to its stable physical properties and high abrasion resistance. Additionally, PEEK can be more aesthetic than metal and conventional thermoplastic resins, enabling its use in the field of dentistry for applications such as implant bodies, implant superstructures, crowns, and fixed partial dentures [1,2,3]. Recently, the use of PEEK as the clasp of removable partial dentures has also been proposed [4,5]. PEEK clasps would be able to reduce the excess force on the abutment teeth, as the elastic modulus of PEEK is approximately 4 GPa. Additionally, low specific weight and high resistance to plaque accumulation, as well as no metallic appearance and allergic factors are preferable for removable partial dentures [6].

Nevertheless, the PEEK material must be strongly affixed to the acrylic resin when using it as a component of dental prosthesis. Acrylic resin is used as a repair material in fixed prostheses, as well as a denture base material. The adhesive strength between the PEEK and acrylic resin significantly influences the probability of denture fracture. Many studies have analyzed the adhesive strength of metal surface treatments to denture base resins [7,8,9,10,11,12,13,14,15,16], suggesting that such treatments lead to improvement in the bending strength of the denture, while providing protection from microleakage between the interface, discoloration, and denture fracture. Studies on the bond strength of PEEK to luting cement or veneering resins for fixed prostheses have been reported [17,18,19,20,21,22,23,24,25]; however, information on the bonding strength between PEEK (applied to the clasp) and acrylic resin is lacking. Other prostheses made of PEEK, such as implant superstructures, crowns, and fixed partial dentures occasionally require fixation using acrylic resin; hence, information on the bond strength of PEEK to acrylic resins is required.

The purpose of this study was to evaluate the shear bond strength of PEEK to acrylic resins, to enable the application of PEEK to the clasp in removable dentures and the fixation of PEEK prostheses, by proposing an effective surface treatment for PEEK to enhance its bond strength.

## 2. Materials and Methods

### 2.1. Materials and Specimen Preparation

The materials used for the shear bond strength tests in this study are shown (Table 1). The adherent materials were PEEK and a cobalt-chromium (Co-Cr) alloy, and the adhesive materials were two types of autopolymerizing resins. Surface treatments were: Al_2_O_3_ sandblasting, tribochemical silica airborne-particle abrasion (Rocatec system), and ceramic or metal primer applications.

The specimens were disks of 15 mm diameter and 3 mm height. PEEK discs were designed on a computer and machine-milled (RXP500 DSC, Roeders BmbH, Soltau, Germany). Co-Cr alloy discs were prepared using a conventional cast technique. Thereafter, all specimens were manually trimmed, polished under water with 600-grit silicon carbide (SiC) paper, and ultrasonically cleaned in distilled water for 10 min.

### 2.2. Surface Treatments

The sample groups were divided into:

- NT: No treatment.
- CP: Priming with the ceramic primer by using a dental microbrush and air-drying.
- SB: Sandblasting with 50 μm Al_2_O_3_ under pressure (0.3 MPa) for 10 s at a distance of 10 mm, with a vertical angle between the nozzle and specimen surface. This was followed by cleaning ultrasonically for 10 min in distilled water and air-drying [25].
- RC: Treating with the Rocatec system, which involved sandblasting with 110 μm SiO_2_-coated Al_2_O_3_ under pressure (0.3 MPa) for 13 s at a distance of 10 mm, with a vertical angle between the nozzle and specimen surface, followed by air-drying of approximately 5 s (with the confirmation of surface condition) [26].
- RCC: Treating with Rocatec (as described above) followed by air-drying and then priming with the ceramic primer by using a dental microbrush and air-drying.
  The Co-Cr surfaces were treated as follows:
- MP: Priming with the metal primer by using a dental microbrush and air-drying.
  10 specimens were fabricated for every condition.

### 2.3. Shear Bond Strength Test

**Figure 1:**
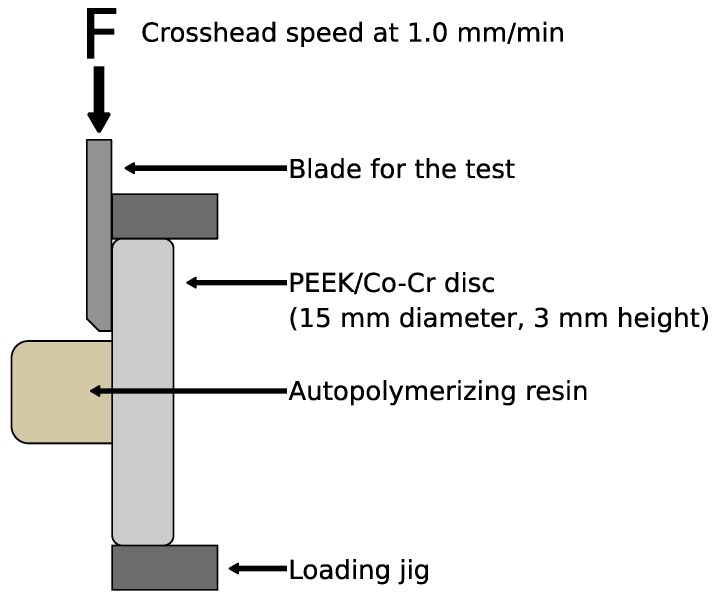
Shear bond strength measurement.

The setup for the shear bond strength test is illustrated in Figure 1.

All specimens were allocated to one of the two bonding resins. A silicone rubber hollow cylinder measuring 5 mm in inner diameter and 5 mm in thickness (Labocone putty, GC Corp., Tokyo, Japan) was attached to the bonding surface of each specimen with silicone putty. An autopolymerizing resin (Unifast II: mixed in powder/liquid ratio of 2 g/1.5 mL) was poured into the cylinder. Unifast II was then polymerized at room temperature (approximately 27 °C) for more than 3 min. The other autopolymerizing resin (Palapress Vario: mixed in powder/liquid ratio of 5 g/3.5 mL) was filled in the same manner. Polymerization was performed according to the manufacturer’s instructions, in a pressure-curing unit (Palamat practic EL T, Kulzer GmbH, Hanau, Germany) at 55 °C and 0.2 MPa pressure for 30 min. The specimens attached with the resin were taken out from the pressure-curing unit and cooled at room temperature. Thereafter, the specimens were carefully removed from the cylinder in a slow manner by hand. All specimens were stored in distilled water at 37 °C for 24 h before testing.

Each specimen was mounted with a special loading jig on a universal testing machine (Autograph AG-X, Shimadzu Corp., Kyoto, Japan). The shear bond strength was evaluated at a crosshead speed of 1.0 mm/min with a load cell of 1 kN. The force at which the bond failed was recorded, and the bond strength was calculated in MPa. The measurements were repeated ten times in every condition.

The cross-sectional surfaces corresponding to bond failure were visually observed with a stereoscopic microscope (Optiphot, NIKON Instech Co., Ltd., Tokyo, Japan) under low-power magnification (100 ×).

### 2.4. Data Presentation and Statistical Analysis

Means and standard deviations of the shear bond strength were calculated in every condition. Firstly, the means of the PEEK groups were analyzed by two-way ANOVA, followed by Bonferroni’s multiple comparison tests at 0.05 significance levels. The highest value in the PEEK treatments and the value for the Co-Cr alloy were compared by the student’s *t*-test (*p* < 0.05). The data were analyzed with a statistical software package (IBM SPSS Statistics version 24.0, IBM Japan, Ltd., Tokyo, Japan).

## 3. Results

**Figure 2:**
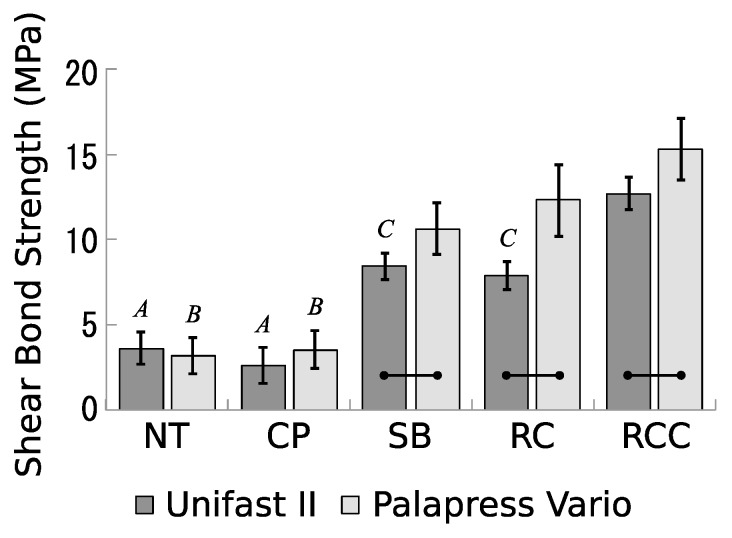
Means and standard deviations of shear bond strengths for surface-treated polyetheretherketone (PEEK) and bonded resins. Means with the same uppercase letters indicate no significant difference (*p* > 0.05) in the treatments. Means with bars indicate a significant difference (*p* < 0.05) in the two resins.

Regarding the bond strength of PEEK to Unifast II and Palapress Vario, means and standard deviations of the shear bond strength are shown (Figure 2). Two-way ANOVA suggested that there were significant differences between treatment groups (*F* = 265.16, *p* < 0.001), between resin groups (*F* = 56.59, *p* < 0.001), and the interaction between both groups (*p* < 0.001).

The bond strengths of NT and CP in Unifast II were 3.61 ± 0.95 MPa and 2.61 ± 1.05 MPa, respectively, and no significant differences were found. The bond strengths of SB and RC in Unifast II were 8.45 ± 0.80 MPa and 7.89 ± 0.85 MPa, respectively, and no significant differences were found. The bond strengths of SB and RC in Unifast II were significantly higher than those of NT and CP. The bond strength of RCC in Unifast II was 12.71 ± 0.94 MPa, and was significantly higher than those for the other four treatments.

The bond strengths of NT and CP in Palapress Vario were 3.19 ± 1.06 MPa and 3.55 ± 1.14 MPa, respectively, and no significant differences were found. The bond strengths of SB and RC in Palapress Vario were 10.63 ± 1.53 MPa and 12.31 ± 2.10 MPa, respectively, and no significant differences were found. The bond strengths of SB and RC in Palapress Vario were significantly higher than those of NT and CP. The bond strength of RCC in Palapress Vario was 15.32 ± 1.80, and was significantly higher than those for the other four treatments. Comparing the bond strengths to Unifast II and Palapress Vario in every treatment condition, the bond strengths to Palapress Vario in SB, RC, and RCC were significantly higher than those to Unifast II.

**Figure 3:**
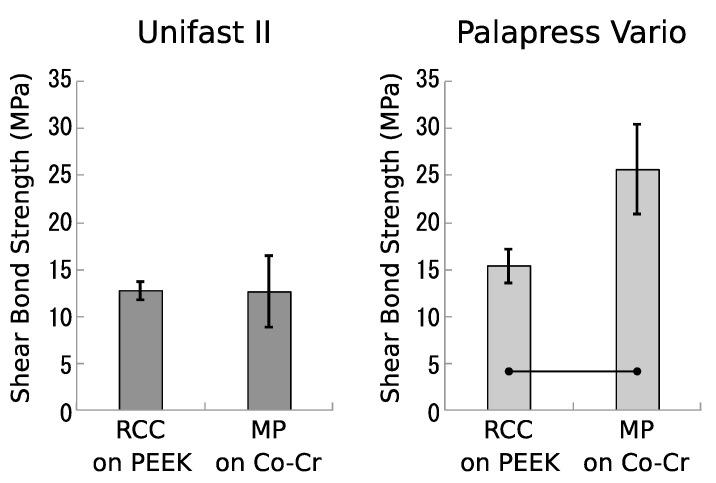
Means and standard deviations of shear bond strengths for PEEK and Co-Cr alloy bonded to resins. Bar indicates a significant difference (*p* < 0.05).

The bond strengths of MP in the Co-Cr and RCC in the PEEK, which showed the highest bond strength in the PEEK materials, are shown (Figure 3). The bond strength of MP to Unifast II was 12.42 ± 3.60 MPa, and was similar to that of RCC. The bond strength of MP in the Palapress Vario was 25.67 ± 4.77 MPa, and was significantly higher than that of RCC.

**Figure 4:**
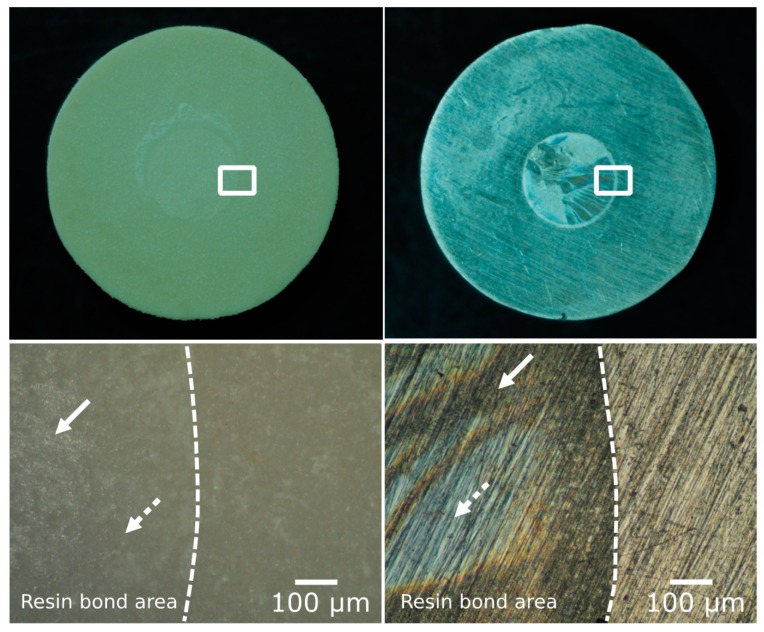
Failure cross-section (stereoscopic observations, 100×). **Left**: PEEK with Rocatec and ceramic primer (RCC) bonded to Palapress Vario, **Right**: Co-Cr alloy with metal primer bonded to Palapress Vario. The dashed line shows a boundary between the resin bond area and non-bond area. The arrow indicates resin remaining on the surface. The dotted arrow indicates no resin remaining on the surface.

Stereoscopic microscope observations of the failure cross-section suggested that mixed adhesive/cohesive failures occurred in SB, RC, and RCC treatments of PEEK bonded to Palapress Vario and Co-Cr alloy specimens, whereas adhesive failures were observed in other treatments of PEEK (Figure 4).

## 4. Discussion

PEEK possesses an aromatic molecular backbone with combinations of ketone (−CO−) and ether (−O−) functional groups between the aryl rings, and is a dominant member of the poly-aryl-ether-ketone (PAEK) polymer family [27]. PEEK has excellent thermomechanical properties, chemical stability, and hydrolysis resistance compared to other plastic materials [4]. PEEK is also a very stable polymer in that it cannot be dissolved by any alkali or acid (with the exception of sulfuric acid). Hence, it is difficult to treat its surface chemically by using general etching agents commonly used in the field of dentistry. Several studies have recommended sulfuric acid etching, air abrasion, or air abrasion with piranha acid etching [28,29]. However, sulfuric acid alters the chemical characteristics of PEEK, owing to the presence of oxygen in the acid, whereas piranha acid breaks the benzene ring [20]. It has been reported that such treatment increases the functional bonding potential of the surface [17,25]. However, PEEK component modification using etching agents at the chairside poses a safety risk. Therefore, five kinds of treatments suitable in a chairside setting were investigated with surface treatments of PEEK. PEEK treated with both Rocatec and ceramic primer had higher bond strengths to both Unifast II and Palapress Vario. According to the manufacturer’s scientific product profile of the Rocatec system, when SiO_2_-coated 110 μm aluminum oxide is blasted onto the surface, it provides a rough surface and fine triboplasma formation on the blasting surface. The SB and RC treatments with sandblast treatment had significantly higher bond strength compared to the control (NT) due to the mechanical interlocking of the PEEK and acrylic resin. Thus, there were no significant differences between the two. Moreover, RCC, in which the surface treated with Rocatec was applied with the ceramic primer, enhanced more bonding strength. All results considered, the highest bonding strength of RCC may be explained by the mechanism in which SiO_2_-coated 110 μm aluminum oxide particles remained on the treated surface by Rocatec, and how the surfaces of particles were treated by a silane coupling agent [14,26].

The bond strength to Palapress Vario proved to be higher than that of Unifast II. Reasons for this could be the difference between the resin material flow and the degree of polymerization [30]. Palapress Vario, with the higher-flow resin, can spread into the texture details of a rough surface. The bond strength of PEEK basically depends on the mechanical locking, which is supported by the following findings; the bond strength of NT or CP without mechanical locking showed a similar value in both Unifast II and Palapress Vario, whereas conversely, the bond strength of SB, RC, and RCC with mechanical locking showed a significant difference between Palapress Vario and Unifast II. In addition, the fact that Palapress Vario used a pressure-pot curing method compared to the bench curing method of Unifast II suggests that Palapress Vario has a higher degree of polymerization [8].

Although the bond strength of Co-Cr alloy to the autopolymerizing resin was set as a target for the bond strength of PEEK, the maximum bond strength of PEEK in any treatment was similar to that of Co-Cr alloy in Unifast II and approximately 60% that of Co-Cr alloy in Palapress Vario. This may be because the particle diameters of sandblast in Co-Cr alloy and PEEK materials were 50 μm and 110 μm, respectively. The low flow Unifast II might not spread to the surface texture of 50 μm, whereas high-flow Palapres Vario might spread to the surface. It was reported that the Rocatec silica particles loosely covered the PEEK surface, and silica particles can easily be lost [22,26]. As a result, RCC might show lower bond strength than that of Co-Cr.

Matsumura et al. reported that, on the basis of clinical research, the bond strength of resin composite veneering materials to gold alloy should be greater than 10 MPa [31]. NaBadalung and Powers reported that the Ni-Cr-Be alloy treated by Met-Etch (metal etching gel) or Rocatec yielded a high bond strength (about 19 MPa) to denture base resins, and that the bond strength could be worthy of clinical evaluation [14]. The bond strength of PEEK to autopolymerizing resins, such as Palapress Vario and Unifast II, after the combination of Rocatec treatment and ceramic priming (RCC), meets the first requirement (>10 MPa), and shows promise for clinical use when compared to the work of NaBadalung and Powers.

Although thermocycling to evaluate the sequential changes of bond strength should be undertaken, the thermal expansion coefficient of PEEK is near to that of the denture base materials (and negligible compared to that of metal materials), and it is hypothesized that its influence on bond strength degradation may be small. Finally, it should be noted that the retention of the clasp in the denture base resin is possible by means of mechanical locking, due to the clasp-retaining element shape and chemical bonding due to surface treatment of the clasp-retaining element. Given the elastic modulus of PEEK compared to metals, the clasp-retaining element for a PEEK clasp may need to be thicker and wider than that of conventional metal clasps.

## 5. Conclusions

Within the limits of the present study, Rocatec treatment, combined with ceramic primer application, showed the highest shear bond strength of PEEK to both Unifast II and Palapress Vario. The bond strength of PEEK to Unifast II was similar to that of Co-Cr alloy, and 60% to that of Palapress Vario. It was suggested that the treated PEEK could be available for the clasp of removable dentures regarding the adherence to the autopolymerizing resin of the denture base.

## Figures and Tables

**Table 1:** Materials used.

Material		Brand Name	Manufacturer	Lot Number
Adherent materials	PEEK	JUVORA DENTAL DISCS	Invibio Biomaterial Solutions, Lancashire, UK	Not registered
	Cobalt-chromium (Co-Cr) alloy	AICHROM shot type	IDS, Osaka, Japan	00121
Bonding materials	Auto polymerizing resin	Unifast II	GC Corp., Tokyo, Japan	Powder: 1503041 Liquid: 170591
		Palapress Vario	Kulzer GmbH, Hanau, Germany	Powder: R010026 Liquid: R010024
Surface treatment applications	Tribochemical silica airborne-particle abrasion	Rocatec Plus	3M ESPE, St. Paul, MN, USA	613946
	Ceramic primer	Clearfil Ceramic Primer Plus	Kuraray Noritake Dental Inc., Tokyo, Japan	9V0020
	Metal primer	Metal Primer Z	GC Corp., Tokyo, Japan	1612152
